# Supplementary material for: A Global Screen for Assembly State Changes of the Mitotic Proteome by SEC-SWATH-MS
Source: Cell Syst. 2020 Feb 26;10(2):133–155.e6. doi: 10.1016/j.cels.2020.01.001 (PMC7042714; doi:10.1016/j.cels.2020.01.001)

Q9BXS4 | TMM59_HUMAN | TMEM59 C1orf8 HSPC001 UNQ169/PRO195
Monomer MW [kDa]: 36.223 Monomer expected elution fraction: 49

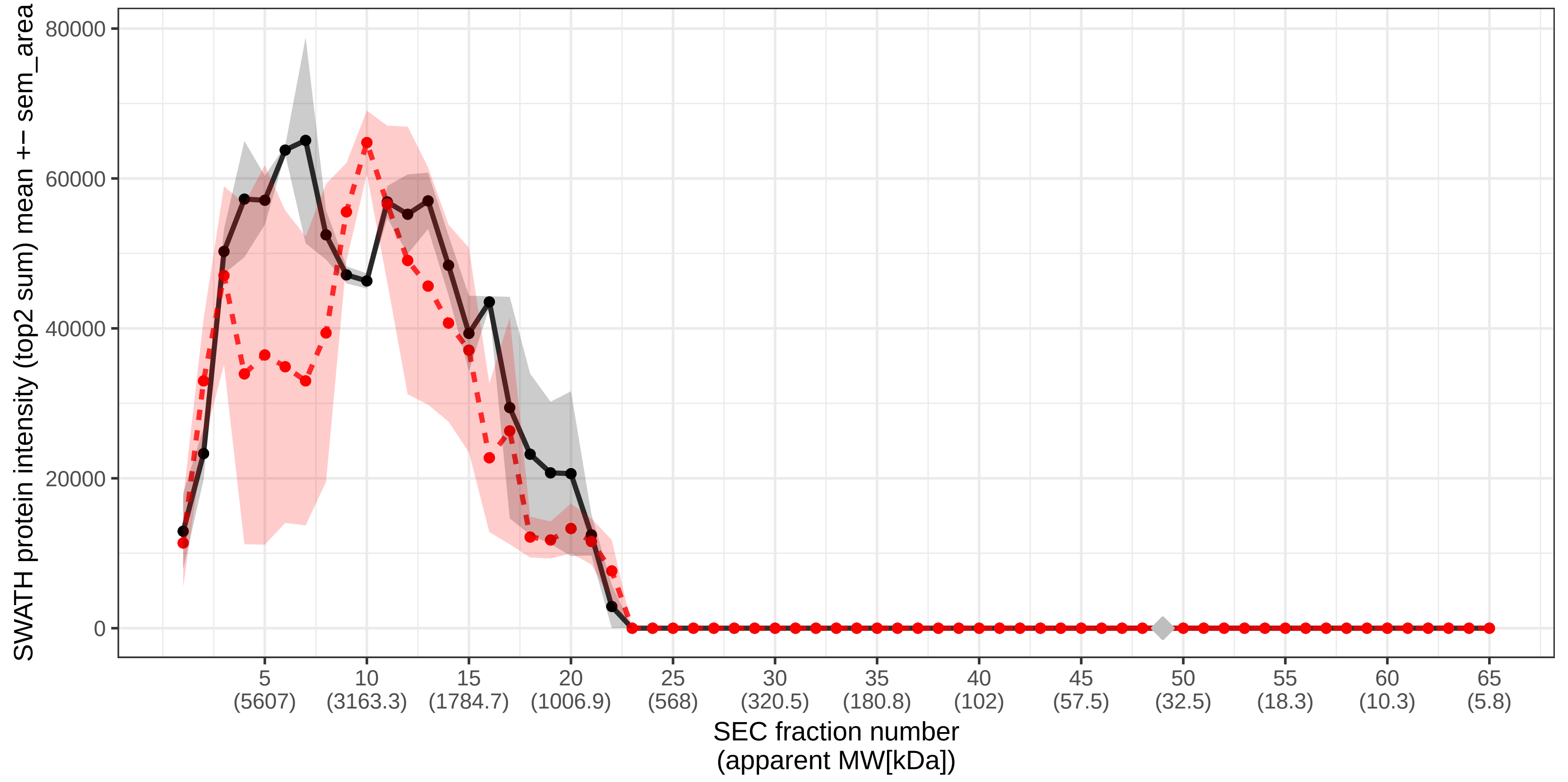

Supplement: Data S1. SEC-SWATH-MS Protein Chromatograms, Related to Figure 1 [file mmc6.zip › SECchrom_Q9BXS4_TMM59_HUMAN_TMEM59_C1orf8_HSPC001_UNQ169_PR.pdf]
